# Effects of Cold Deformation and Heat Treatments on the Microstructure and Properties of Fe-15Cr-25Ni Superalloy Cold-Drawn Bars

**DOI:** 10.3390/nano14231949

**Published:** 2024-12-04

**Authors:** Yunfei Zhang, Zhen Zhang, Zhiyan Sun, Yingli Zhao, Yi Cui, Zhongwu Zhang

**Affiliations:** 1 School of Materials Science and Chemical Engineering, Harbin Engineering University, Harbin 150001, China; 2 HBIS Group, HBIS Materials Technology Research Institute, Shijiazhuang 050023, Chinasunzhiyan@hbisco.com (Z.S.); cuiyi@hbisco.com (Y.C.)

**Keywords:** cold-drawn, solid solution, aging treatment, Fe-15Cr-25Ni superalloy

## Abstract

The combination of cold deformation and solution aging is an important technological route for the bar processing of superalloy fasteners. The microstructure evolution and mechanical properties are intimately related to the process parameters. In this study, we systematically elucidate the mechanical properties and microstructure evolution of Fe-15Cr-25Ni alloys in different treatment processes and conduct in-depth analysis of the synergistic strengthening mechanism of fine-crystal strengthening, second-phase strengthening, and work hardening on Fe-15Cr-25Ni alloys. The results show that the tensile strength and yield strength at room temperature increase with the increase in grain refinement and dislocation density but decrease with the increase in elongation. After solid-solution treatment, most of the precipitates dissolve into the matrix, and the dislocation density is greatly reduced, resulting in a decrease in strength and an increase in plasticity. After aging, a large amount of γ′ phase was precipitated. Due to the two strengthening effects of dislocation strengthening and second-phase strengthening, the strength of the aging state is more improved than that of the cold-drawing state. The purpose of this study is to provide valuable insights for the industrial production of Fe-15Cr-25Ni superalloys.

## 1. Introduction

The Fe-15Cr-25Ni superalloy demonstrates high yield strength and outstanding durability at temperatures approximately 650 °C, thus being widely employed in the manufacturing of high-temperature bearing components for aircraft engines, such as turbine disks, press disks, rotor blades, and fasteners. The alloy is strengthened through a spherical ordered face-centered cubic (FCC) γ′ phase [1,2,3,4,5,6,7].

At present, a large number of studies focus on the traditional thermal deformation and heat treatment behavior of Fe-15Cr-25Ni alloy [8,9,10], and the influence of cold deformation on the alloy is less studied. Huang et al. [11] identified a new high pre-strain twining–detwinning process, which enabled the studied alloy to obtain an ultra-high yield strength of 967 MPa and a reasonable elongation of 12%. Liu Jimeng et al. [12] conducted an experimental study on the defects of mixed-crystal strips in cold-drawn Fe-15Cr-25Ni alloy bars, revealed the main causes of the mixed-crystal strip, and analyzed its internal microstructure and the influence of the mixed-crystal structure on microhardness. Liu Sicheng et al. [13] studied the changes in grain morphology, precipitated phase, and tensile properties of the alloy under different shape variables. The results showed that when the deformation amount was less than 30%, the alloy structure was equiaxed and there were more twins and when the shape variable was more than 35%, the structure began to experience fibrosis and obvious deformation characteristics appeared in the crystal. For the same aging system, the amount of dispersed γ′ phase, granular titanium carbide, and chromium carbide precipitates increases with the increase in the shape variable, and the tensile strength of the alloy also increases.

The research of Fe-15Cr-25Ni alloys in the field of cold deformation and heat treatment has accumulated relatively rich results, but the exploration of its cold deformation behavior and the systematic effects of subsequent heat treatment on the microstructure and macro properties of materials are still in the stage of urgent deepening. In view of this, the preparation technology of cold-drawn Fe-15Cr-25Ni alloy bars was discussed. The mechanical properties and microstructure evolution of hot-rolled solid-solution Fe-15Cr-25Ni alloy bars after cold drawing, solution, and aging treatments were studied in this paper, aiming to provide valuable insights on how to accurately control process parameters and stably improve product quality in practical industrial production.

## 2. Materials and Methods

The chemical composition of the Fe-15Cr-25Ni alloy is presented in Table 1. The φ15 mm cold-drawn billet was fabricated via a vacuum induction melting process, electroslag remelting process, forging, rolling, and solution treatment.

The cold-drawing test of the hot-rolled annealed bar with a diameter of 15 mm was conducted by a double-chain drawing machine. The annealing system was 900 °C for 1 h followed by water cooling. The cold-drawing test was carried out on the hot-rolled annealed bar with the double-chain drawing machine. The total cold-drawing deformation was 50%. A horizontal annealing furnace was used for the solid-solution treatment of cold-drawn bars, with a solid-solution state maintained at 900 °C for 2 h followed by water cooling. Then, the solid-solution bars were aged, and the horizontal annealing furnace was used for 16 h and air cooling at 700 °C and 16 h and air cooling at 650 °C. Finally, samples in four different states, namely hot-rolled solid solution, cold drawing, solid solution, and aging, were obtained, as shown in Table 2. Then, tensile properties, hardness, microstructure, and precipitated phase were tested at room temperature. The chemical analysis method was X-ray fluorescence spectrometry (XRF). The tensile properties of the alloy were tested using the Z600E-600N electronic tensile testing machine manufactured by Zwick Company (Ulm, Germany) at a strain rate of 0.00025s ^−1^. The tensile samples were prepared with a CNC lathe according to GB/T 228.1-2010 standard [14]. The Vickers hardness (HV) was determined using a Williams-type Tukon 2500 fully automatic Vickers hardness tester, with at least 3 measurements taken at different points on each sample to obtain the average hardness value. The samples were observed by optical microscopy (OM) and scanning electron microscopy (SEM) using mechanical polishing and etching (4%). The equipment used for the SEM was a Hitachi S-3400N (Tokyo, Japan) scanning electron microscope, the acceleration voltage was set to 20 KV, the measurement mode was a secondary electron mode, and the microstructure of materials was studied. The texture and dislocation density of the materials were studied with a directional imaging microscope (EBSD) on the Hitachi S-3400N scanning electron microscope. A PANalytical X’ Pert Pro X-ray diffractometer (Malvern, UK) with cu-k-α radiation was used to analyze the phase composition of the sample. The 20°~90°2θ Angle was scanned at 40 KV, and the step was 0.02°. The sample was a 20 mm long cylinder, the center line of the extended cylinder was cut lengthwise, and the detection plane was longitudinal section. XRD data were analyzed using Highscore v. 3.0.5 software (Malvern PANalytical, Malvern, UK). The precipitated phase was analyzed with a Tecnai F30 transmission electron microscope (TEM, Hillsboro, OR, USA). The acceleration voltage was 300 KV, and the sample was mounted with a double-tilting rod. The measurement modes included bright field, dark field, EDS spectrum, and selected electron diffraction (SAED).

## 3. Results and Discussion

### 3.1. Mechanical Properties

**Figure 1:**
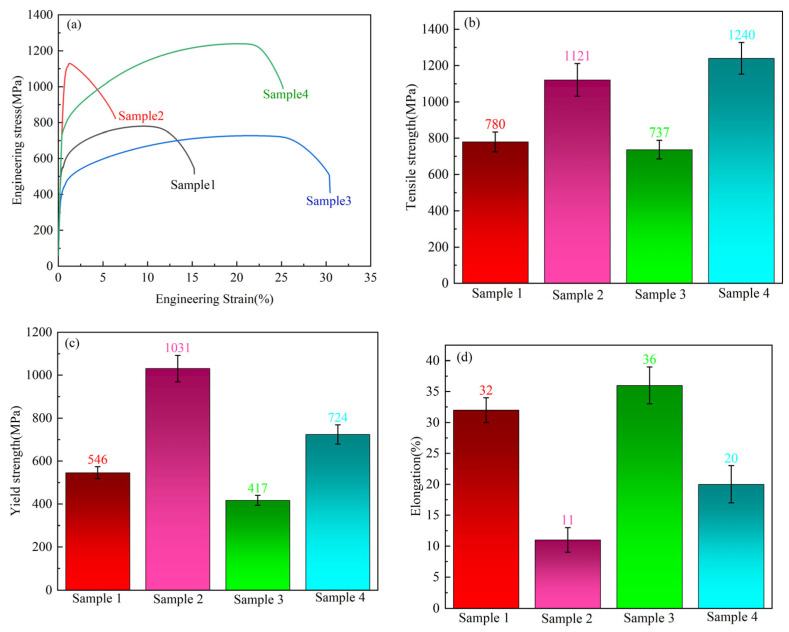
Tensile properties of Fe-15Cr-25Ni samples under different states at room temperature: (**a**) stress–strain curves, (**b**) tensile strength, (**c**) yield strength, (**d**) elongation.

Figure 1 shows the test outcomes of tensile properties of Fe-15Cr-25Ni samples in diverse states at room temperature. The tensile strengths of the hot-rolled and cold-drawn states are 780 MPa and 1121 MPa, respectively, signifying an increase in tensile strength of approximately 43%. The yield strengths are 546 MPa and 1031 MPa, respectively, indicating an increase of approximately 88%. Nevertheless, the elongation reduces from 32% to 11%. The Fe-15Cr-25Ni alloy demonstrates a high-strength and low-plasticity characteristic after cold drawing.

The determining factors for the performance of the alloy after cold drawing are mainly in the following two aspects:

(1) The intrinsic nature, lattice type, and state of the alloy are characterized by the deformation-strengthening index.

Fe-15Cr-25Ni alloy is an iron–nickel-based austenitic superalloy; the main chemical composition includes iron, nickel, chromium, and added molybdenum, titanium, aluminum, and vanadium to enhance its comprehensive properties. The alloy has a face-centered cubic structure.

According to the logarithmic form of Hollomon Equation [15]:

lg*S* = lg*K* + *n*lg*ε* (1)

where *S* represents the true stress, *K* is the hardening coefficient, *n* is the deformation strengthening index, and *ε* is the true strain.

**Figure 2:**
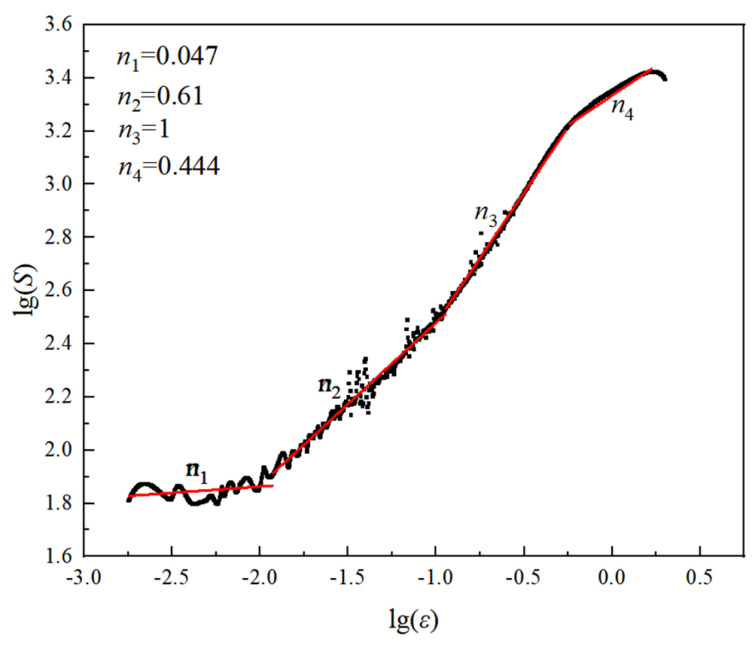
Room temperature lg*S*−lgε curve.

According to Equation (1), a plot of lg*S*-lg *ε* is drawn, as shown in Figure 2, with a slope of *n*. Deformation strengthening of Fe-15Cr-25Ni alloy from yield to fracture can be divided into the following: yield-deformation-strengthening stage *n*_1_, pre-uniform-deformation-strengthening stage *n*_2_, post-uniform-deformation-strengthening stage *n*_3_, and local-set-plastic-deformation-strengthening stage *n*_4_. After averaging the *n* values of the four strengthening stages, the cold-drawn deformation-strengthening index is 0.53. The results show that Fe-15Cr-25Ni alloy has good cold deformation workability and excellent uniform deformation ability.

(2) The reduction ratio during cold drawing. The tensile strength of the rod after cold drawing can be approximately expressed by Equation (2) [16,17]:
  *σ* = *σ_m_* − 10,580*z*^3^ + 4776*z*^2^ + 613*z* (2)
  where *σ* represents the tensile strength of the rod after cold drawing. *σ*_m_ is the tensile strength of the hot-rolled rod (after solution treatment). *z* is the reduction ratio. According to Equation (2), the calculated tensile strength after a 50% cold-drawing deformation amounts to 958 MPa. Nevertheless, the experimental result is 1121 MPa, which may be ascribed to differences in the alloy’s cold-drawing rate and the deformation amount per pass.

The tensile strength and yield strength of the solid-solution state are 737 MPa and 417 MPa, respectively, with the elongation at 36%. In contrast to the cold-drawn state, there is a considerable reduction in both tensile strength and yield strength, while the elongation increases significantly. Following aging, there is a notable increase in both tensile strength (1240 MPa) and yield strength (724 MPa), yet the elongation drops to 20%.

**Figure 3:**
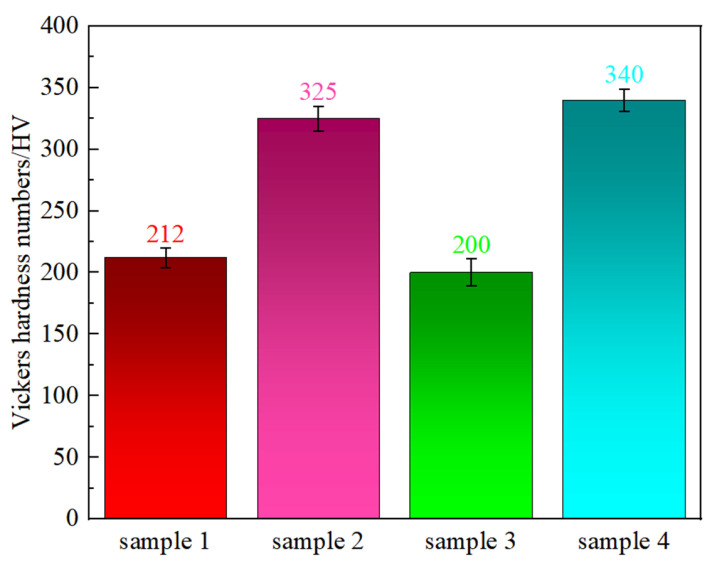
The Vickers hardness of the Fe-15Cr-25Ni alloy with different preparation processes.

Figure 3 shows the Vickers hardness of the Fe-15Cr-25Ni alloy. The Vickers hardness of the hot-rolled samples is 212 HV. Following cold drawing, the hardness of the sample rose from 212 HV to 325 HV, with an increase of 53%. After the subsequent solution treatment, the hardness dropped to 200 HV, a reduction of approximately 63%. Subsequent aging treatment markedly increased the hardness to 340 HV, an increase of approximately 70%. Additionally, the Vickers hardness of the aged state is higher than that of the cold-drawn state, which is similar to the change trend in the tensile strength, as depicted in Figure 1.

### 3.2. Microstructure

**Figure 4:**
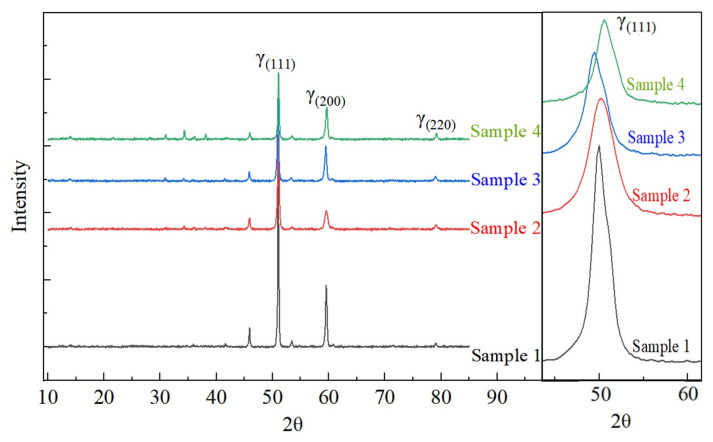
The XRD diffraction patterns of the Fe-15Cr-25Ni alloy with different preparation processes.

The XRD diffraction patterns of the Fe-15Cr-25Ni specimens in different states are presented in Figure 4. The XRD patterns of all four states exhibit considerable similarity, with no distinguishable emergence of new peaks. A comparison with the standard cards for the γ-(Fe, Ni) phase and the γ′-Ni_3_(Ti, Al) phase reveals that all four states present a sole γ-(Fe, Ni) austenite phase. After aging, no distinct diffraction peaks corresponding to a secondary phase in the XRD patterns can be discerned due to the low concentration and weak diffraction intensity of the second phase. Nevertheless, a notable rightward shift can be observed in the XRD pattern of the aged samples, suggesting a reduction in the lattice constant of the matrix material in accordance with Bragg’s equation (2*d*sin*θ* = *nλ*). During early stages of aging treatment, precipitated phases typically maintain either coherent or semi-coherent relationships with the matrix phase. Hence, their lattice constants differ from that of γ-Fe and contribute to lattice distortion upon their accumulation. Consequently, extrusion of these precipitates leads to a decrease in the lattice constant of γ-Fe and results in the observed rightward shift in its diffraction peak position [18,19,20]. It is notable that due to the low-volume fractions of precipitates, characteristic peaks corresponding to specific precipitate phases cannot be detected merely by XRD analysis. The absence of the characteristic peaks for the second phase does not signify the non-existence of the second-phase particles after aging treatment within this series of alloy systems. This conclusion is further substantiated by subsequent microstructural characterization results.

**Figure 5:**
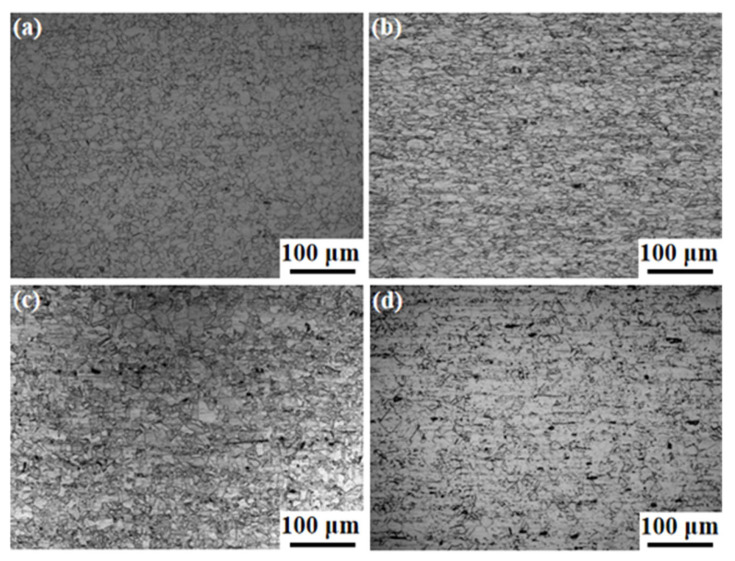
The OM microstructure of the Fe-15Cr-25Ni alloy: (**a**) Sample 1; (**b**) Sample 2; (**c**) Sample 3; (**d**) Sample 4.

Figure 5 shows the OM microstructure of the Fe-15Cr-25Ni in different states. Figure 5a depicts the microstructure of the hot-rolled specimen, which is characterized by a homogeneous grain structure and equiaxed distribution, with an average grain size of 12 μm. Figure 5b shows the cold-drawn structure of Fe-15Cr-25Ni, the changes in grain shape and elongation along the cold-drawn direction, and more twins were found. Figure 5c presents the microstructure of a cold-drawn bar after solid-solution treatment, disclosing bands consisting of coarse and fine crystals due to the limited solution time and insufficient grain growth. Maximum diameters of coarse grains can reach 40 μm and fine grains down to 7 μm. Figure 5d shows the aging microstructure which exhibits minimal changes in grain size after post-aging treatment. Grain growth is mainly influenced by two major factors: one is a driving force for grain boundary movement originating from the decrease in the interfacial energy, and the other one is the reduced mobility at these boundaries during solid-solution treatments.

**Figure 6:**
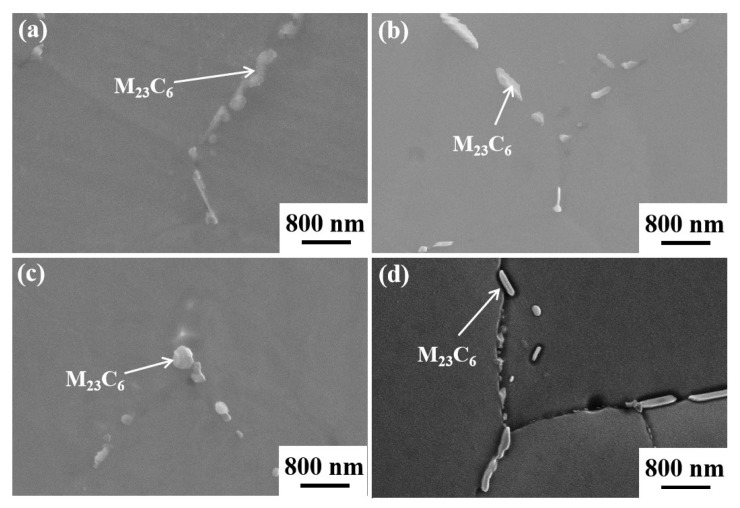
SEM maps of Fe-15Cr-25Ni alloy: (**a**) sample 1; (**b**) sample 2; (**c**) sample 3; (**d**) sample 4.

**Figure 7:**
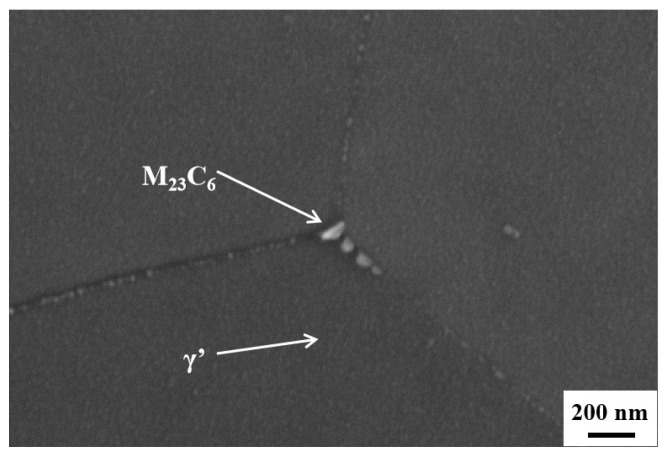
SEM maps of the aged Fe-15Cr-25Ni alloy.

Figure 6 shows the SEM images of the Fe-15Cr-25Ni alloy in different states, and the detection position is 1/2R. Precipitates were observed on the grain boundaries of all four samples. EDS analysis revealed that these precipitates were identified as the M_23_C_6_ phase, as indicated in Table 3. In the aged state, a large quantity of fine precipitated phase, namely the γ′ phase, can be identified within the grains (Figure 7). The spherical γ′ phase is uniformly distributed within the grains, with a small amount at the grain boundaries. Additionally, M_23_C_6_ phases are also observed at the trinomial grain boundaries. This observation further accounts for why the XRD analysis fails to detect obvious diffraction peaks from these second phases.

**Figure 8:**
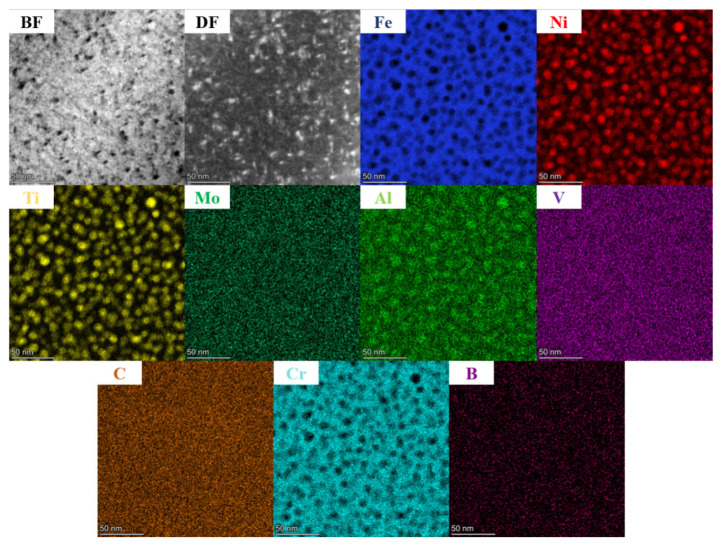
TEM-EDS diagram of aged Fe-15Cr-25Ni alloy.

**Figure 9:**
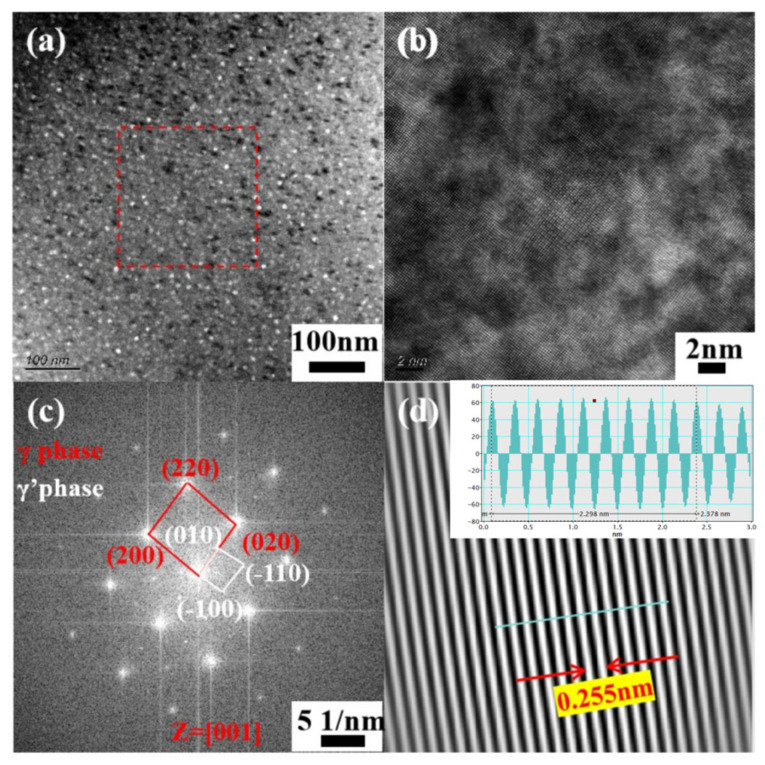
TEM images of aged Fe-15Cr-25Ni alloy: (**a**) local bright field image, (**b**) local high-resolution image, (**c**) Fourier transform image, (**d**) inverse Fourier transform image.

In order to further clarify the morphology and composition of the precipitated γ′ phase after aging, a TEM analysis was performed on sample 4, and the results are presented in Figure 8. The bright field and dark field images distinctly disclose a spherical precipitated phase with an approximate diameter of 11.6 nm, mainly composed of Ni, Ti, and Al. The high-resolution TEM results are presented in Figure 9. In Figure 9a, a uniform distribution of bright white spots with varying sizes can be witnessed. Local high-resolution observation was carried out on the region enclosed by the red box, followed by Fourier transform applied to the high-resolution map as shown in Figure 9c. The presence of the γ′ phase was identified by the diffraction analysis. Both γ phase and γ′ phase exhibit face-centered cubic structures, with incident axis Z = [001] calculated in accordance with crystal zone axis principles. The inverse Fourier transformation diagram of the γ′ phase is illustrated in Figure 9d. Measurements indicated a crystal plane spacing of 0.255 nm along the (−110) direction for the γ′ phase.

**Figure 10:**
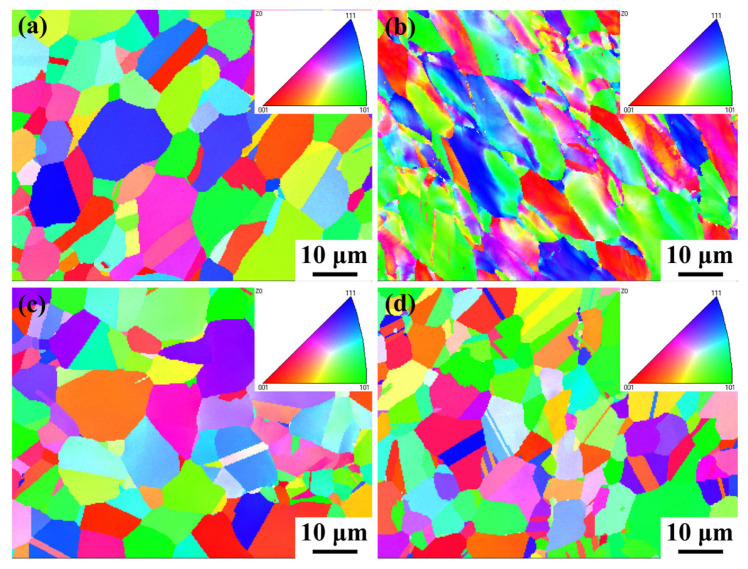
IPF maps of Fe-15Cr-25Ni alloy: (**a**) sample 1; (**b**) sample 2; (**c**) sample 3; (**d**) sample 4.

Figure 10 shows the inverse pole figure (IPF) maps of Fe-15Cr-25Ni in different states. It is noted that, except for sample 2, the grain orientations of other samples are approximately random, showing isotropy. For sample 2, treated by cold drawing, due to the tensile stress, grains are elongated, and grain orientations gradually tend to be uniform. The preferred orientations are <101> and <111>. The <111> orientation is typically the ultimate stable texture component in the tensile deformation of FCC metals. This is because the slip directions of grains oriented along <111> are symmetrically distributed in relation to the axis direction, which facilitates maintaining the circular cross-section of the metal wire during the cold-drawing process. Additionally, the <111> texture can promote the formation of twinning, strengthening the strength of the hard orientation [21,22].

**Figure 11:**
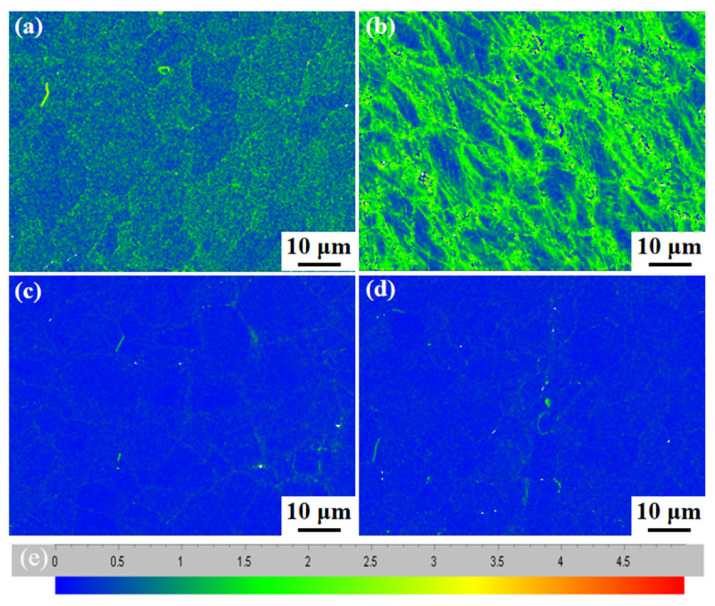
The local misorientation analysis of the Fe-15Cr-25Ni alloy: (**a**) sample 1; (**b**) sample 2; (**c**) sample 3; (**d**) sample 4, (**e**) Color scale.

Figure 11 shows the KAM diagram of Fe-15Cr-25Ni in different states. Figure 11a–d can be evaluated based on the KAM bar of Figure 11e. The location with higher local dislocation value indicates that the plastic deformation degree is larger or the dislocation density is higher; therefore, the dislocation density is the highest after cold drawing [23,24], as shown in Figure 11b. After solution treatment, it can be observed from Figure 11c that the dislocation density of the sample after solution treatment is significantly lower than that of the cold-drawn sample. After aging, as shown in Figure 11d, the dislocation density does not change significantly compared to the sample after solid-solution processing. This implies that the heat treatment process has a significant impact on the dislocation density in the alloy and further elucidates the microstructure and plastic deformation characteristics of the alloy in different states.

**Figure 12:**
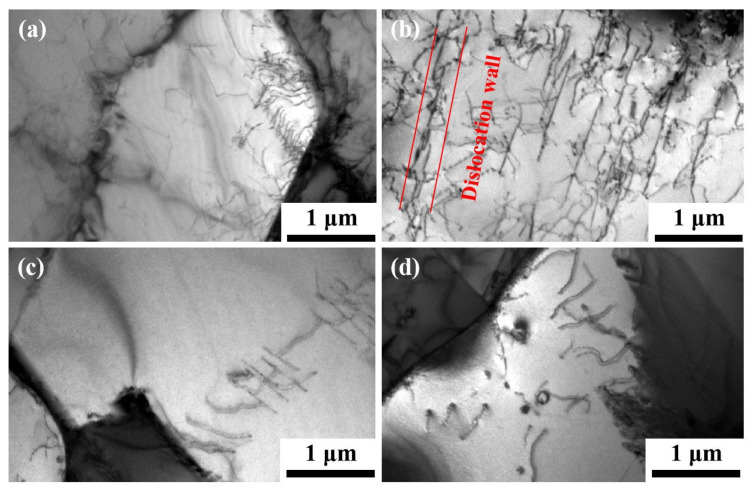
TEM images showing the dislocation morphologies in 4 samples with different states: (**a**) sample 1; (**b**) sample 2; (**c**) sample 3; (**d**) sample 4.

The TEM image in Figure 12 presents the dislocation characteristics of the sample in four distinct states. It is clearly observable that the dislocation slip of hot-rolled solid-solution samples after cold drawing significantly enhances the dislocation density, and multiple dislocation operations lead to dislocation entanglement and the formation of dislocation walls. These walls effectively impede dislocation propagation and contribute to an increase in strength [25,26], as depicted in Figure 12b. After undergoing solution treatment, a significant decrease in placement errors was observed, as depicted in Figure 12c. Dislocations still persist after aging, as demonstrated in Figure 12d. The results obtained through TEM detection are in accordance with those acquired from EBSD detection. The movement of dislocations within grains mainly contributes to grain deformation and is typically impeded by the γ′ phase. When there are few γ′ precipitates and they are relatively distant from each other, dislocation motion becomes more facile along force directions. On the other hand, when there are numerous closely spaced γ′ precipitates within a grain, dislocation motion can be significantly impeded, resulting in reduced deformability and high strength but compromising plasticity characteristics such as elongation capacity [27,28,29]. This observation aligns with room temperature tensile test results.

The strength calculation is according Equation (3) [30,31]:

(3) $$\sigma_{y}=\sigma_{S}+\sigma_{g}+\sigma_{p}+\sigma_{d}$$

where the *σ_s_*—solid-solution strengthening; the *σ_g_*—grain boundary strengthening; the *σ_p_*—precipitation strengthening; the *σ_d_*—dislocation strengthening.

During the cold-drawing process, the degree of deformation increases progressively. The grains gradually decrease, while the deformed grains increase in number. The grains transform from partially equiaxed crystals to completely deformed elongated grains. Simultaneously, the dislocation density gradually increases, indicating macro-level work-hardening phenomenon. Therefore, the improvement in strength is attributed to both fine grains and work-hardening effects, resulting in high strength but low plasticity after cold drawing. Subsequently, the alloy undergoes solid-solution treatment where most of the precipitated phase dissolves back into the solution and significantly reduces the dislocation density, thereby reducing the work-hardening effect and leading to a significant decline in strength. After the aging process, a substantial amount of γ′ precipitates are reduced, leading to the increase in strength by two mechanisms: dislocation strengthening and precipitation strengthening in aged samples.

## 4. Conclusions

This paper probed into the evolution of microstructures, encompassing grain morphology, grain-boundary-segregated second phase, and nanoscale γ′ precipitate during hot rolling, cold drawing, solid solution, and aging. The influences of the microstructure on the mechanical properties are also deliberated. The following conclusions can be drawn:

(1) Under the combined effect of grain refinement strengthening and work hardening during cold drawing, the tensile strength, yield strength, and hardness of the Fe-15Cr-25Ni alloy increased by 43%, 88%, and 53%, respectively, while the elongation decreased by 21%.
(2) After undergoing solid-solution treatment, the majority of the precipitated phase dissolves back into the matrix, and the dislocation density decreases greatly, which leads to the decrease in strength and the decrease in strength.
(3) After the aging treatment of the Fe-15Cr-25Ni alloy, the γ′ phase formed within grains hinders the dislocation movement and increases the strength by precipitation strengthening, leading to the increased strength but decreased plasticity. With dislocation strengthening and precipitation strengthening, the tensile strength reaches up to 1240 MPa, while the yield strength amounts to 724 MPa, with a hardness value of 340 HV and with a satisfied elongation of 20%.

## Figures and Tables

**Table 1:** Chemical composition of Fe-15Cr-25Ni alloy (wt.%).

Elements	C	Fe	Ni	Cr	Mo	Co	Ti	Al	V
Content	0.05	53.90	24.56	15.12	1.26	0.20	1.79	0.19	0.32

**Table 2:** Preparation process of Fe-15Cr-25Ni alloy for testing.

Samples	Preparation Process
1	Hot-rolled annealed (900 °C holding 1 h water cooling)
2	Cold-drawn state
3	Solid-solution state (900 °C holding time 2 h water cooling)
4	Aging state (700 °C holding 16 h air-cooling, 650 °C holding 16 h air-cooling)

**Table 3:** SEM-EDS analysis results.

Elements (at%)	C	Fe	Ni	Cr	Mo	Ti	Al	V
Sample 1	19.7	39.7	23.0	12.3	0.6	3.7	0.5	0.3
Sample 2	21.6	41.0	20.0	13.6	0.6	2.3	0.6	0.3
Sample 3	21.5	42.0	18.6	13.5	0.6	2.5	0.5	0.3
Sample 4	16.0	44.7	20.9	14.4	0.6	2.4	0.6	0.4

## Data Availability

Data are contained within the article.
